# Efficacy of azithromycin in treating *Ureaplasma urealyticum*: a systematic review and meta-analysis

**DOI:** 10.1186/s12879-023-08102-5

**Published:** 2023-03-16

**Authors:** Weibin Fan, Qisheng Wang, Zuyu Liang, Jinyu Wang, Lin Zhang

**Affiliations:** 1 Department of Pharmacy, Changxing People’s Hospital, Changxing, Zhejiang China; 2 Department of Clinical Pharmacy, Xinchang Hospital Affiliated to Wenzhou Medical University, 117 Gushan Middle Road, Xinchang County, 312500 Zhejiang Province China; 3 Department of Hematology, Zhejiang Provincial People’s Hospital, Hangzhou, Zhejiang China; 4 Department of Medical Records and Statistics, The Cancer Hospital of the University of Chinese Academy of Sciences (Zhejiang Cancer Hospital), Institute of Basic Medicine and Cancer (IBMC), Chinese Academy of Sciences, Hangzhou, Zhejiang 310022 China; 5 Department of Clinical Pharmacy, Shaoxing People’s Hospital, Shaoxing Hospital, Zhejiang University School of Medicine, 568# Zhongxing North Road, Shaoxing, 312000 Zhejiang Province China; 6 Key Laboratory of Intelligent Pharmacy and Individualized Therapy of Huzhou, Zhejiang, China

**Keywords:** *Ureaplasma urealyticum*, Azithromycin, Efficacy, Female, Meta-analysis

## Abstract

**Background:**

*Ureaplasma urealyticum* is the most prevalent genital mycoplasma isolated from the urogenital tract of females, but there is no unified treatment plan. This study aimed to evaluate the efficacy of azithromycin in treating *Ureaplasma urealyticum*.

**Methods:**

From the earliest to June 2022, published randomized controlled trials (RCTs) on azithromycin treatment of *Ureaplasma urealyticum* were retrieved by searching PubMed, Embase, Cochrane Library, and Web of Science. Two reviewers independently extracted the data. We utilized the Cochrane risk-of-bias assessment technique to assess the quality of included RCTs. The data were analyzed using the R language (version 4.0.4) software.

**Results:**

Seven RCTs were finally included, involving 512 participants (240 in the experimental group, 272 in the control group). The experimental group was treated with azithromycin monotherapy, while the control group was treated with doxycycline or a placebo. Meta-analysis results suggested that azithromycin has a comparable therapeutic effect on *Ureaplasma urealyticum* in comparison to that of controls (risk ratio [RR] = 1.03, 95% confidence interval [CI] 0.94–1.12). Subgroup analysis showed that the dose and duration of azithromycin may don’t affect its efficacy.

**Conclusion:**

Regarding the meta-analysis that we performed based on existing clinical studies, azithromycin is quite effective in treating *Ureaplasma urealyticum*.

**Supplementary Information:**

The online version contains supplementary material available at 10.1186/s12879-023-08102-5.

## Background

*Ureaplasma* species are the most prevalent genital mycoplasma isolated from the urogenital tract of both men and women [1]. *Ureaplasma* has 14 known serotypes and is divided into two biovars, *Ureaplasma parvum* and *Ureaplasma urealyticum* [2]. Currently, commonly used drugs in *Ureaplasma urealyticum* treatment include quinolones, macrolides and tetracyclines. The growth in the resistance rate of fluoroquinolones, as the first-line clinical drugs, is particularly obvious. Resistance to macrolides and tetracyclines has been reported [3–5]. Azithromycin exhibits high potency against clinical ureaplasma isolates in vitro [6]. However, the clinical efficacy of azithromycin in treating *Ureaplasma urealyticum* remains controversial; therefore, it is necessary to conduct a rigorous systematic evaluation of existing research literature to provide more reliable evidence-based medicine for disease prevention and treatment. It would provide an objective reference for the clinical rationale and scientific use of antimicrobial drugs and the control of *Ureaplasma urealyticum* resistance.

## Methods

This systematic review was registered with the International Platform of Registered Systematic Review and Meta-analysis Protocols (INPLASY) database (registration No. INPLASY202240001).

### Search strategy

The study was conducted following the recommendations of the Preferred Reporting Items for Systematic Reviews and Meta-Analyses (PRISMA) statement [7]. A literature search on PubMed, Embase, Web of Science, and Cochrane Library was conducted, and we limited our search to high-quality studies published until June 2022. The search was undertaken with Medical Subject Headings (MeSH), and appropriate adjustments were made according to the different databases. Search terms included: (1) *Ureaplasma urealyticum* or *Ureaplasma urealyticum biovar 2*, (2) Azithromycin or Azythromycin or Sumamed or Toraseptol or Vinzam or CP-62,993 or CP 62,993 or CP62993 or Zithromax or Azitrocin or Azadose or Ultreon or Zitromax or Azithromycin Dihydrate or Dihydrate, Azithromycin or Azithromycin Monohydrate or Monohydrate, Azithromycin or Goxal or Zentavion. The retrieval search strategy is shown in Additional file 1. The reference lists of the articles identified in the primary search or the relevant reviews identified using the search were also reviewed by three independent reviewers.

### Inclusion/exclusion criteria

The inclusion criteria were as follows: RCT study of azithromycin in treating *Ureaplasma urealyticum* infection in a female reproductive tract over 18 years of age. The exclusion criteria were as follows: (1) letters, reviews, guidelines or editorials; (2) animal studies or cell studies; (3) studies only including the use of azithromycin in males and infants; (4) non-randomized controlled studies; (5) studies in which treatment effect values could not be obtained.

### Data extraction and quality assessment

The following information was extracted by two independent reviewers (Jinyu Wang and Qisheng Wang): author(s), publication year, research design, participant number, types of antibiotics, treatment dose, and mycoplasma cure. Any disagreement on specific studies between the two reviewers was resolved through discussion or consultation with the third reviewer (Zuyu Liang). The quality of the studies was established according to the Newcastle–Ottawa Scale (NOS).

### Risk of bias assessment

Egger’s test [8] was used to exam the publication bias in included study and the revised Cochrane risk of bias tool for randomized trials (RoB2) [9] was applied to assess the risk of bias of RCTs. All risk of bias assessment was assessed by two authors (Weibin Fan and Qisheng Wang) individually and any disagreement was resolved by a third author (Lin Zhang).

### Statistical analysis

The R language (version 4.0.4) software was used for data analysis and visualization. specifically, the meta and metafor packages were used for data processing and forest and funnel plots. Dichotomous variables were analyzed using the odds ratio (OR) and its 95% confidence interval (CI) as statistics for efficacy analysis. Heterogeneity was tested using a Chi-squared test (α = 0.1), combined with a Q test and I^2^ test, and if P > 0.1 and I^2^ ≤ 50%, which suggested less heterogeneity between the studies, a fixed-effect model was used. Otherwise, a random-effect model was used. Significant heterogeneity was addressed via subgroup analysis or sensitivity analysis.

## Results

### Study selection and characteristics

**Fig. 1:**
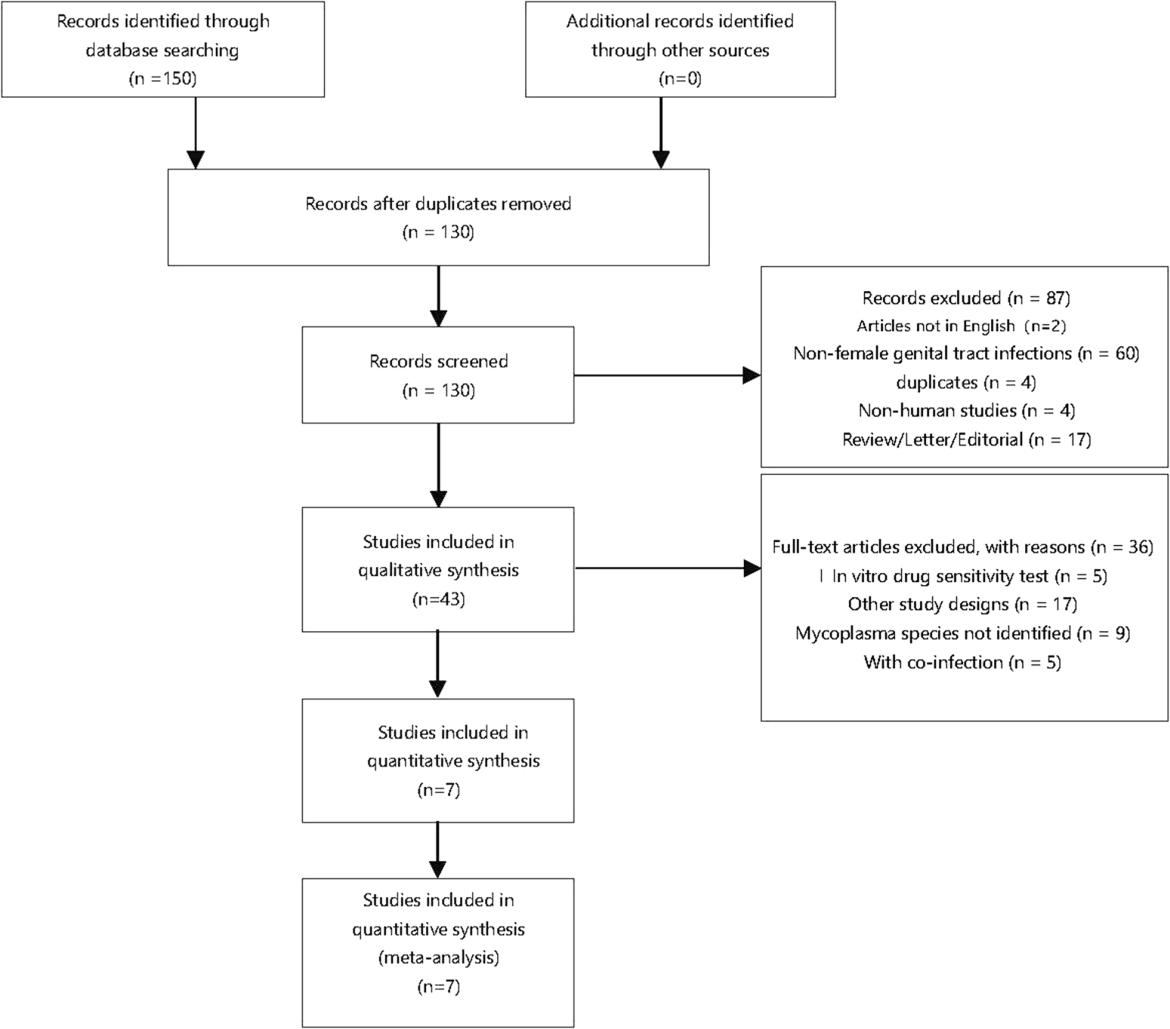
Flow diagram of study selection

Three researchers searched PubMed, Embase, Web of Science, and Cochrane Library and found 83 articles on PubMed, 20 articles on Embase, 44 articles on Web of Science, and 3 articles on Cochrane Library. The search strategy and selection are shown in Fig. 1. Finally, seven studies were included for meta-analysis from the 130 initial research studies after the exclusion strategy. The characteristics of the selected studies are shown in Table 1. These studies included 512 patients with *Ureaplasma urealyticum* (240 patients treated with azithromycin and 272 patients as the control).

**Table 1:** Characteristics of the studies included in the meta-analysis

Study	Region	Year	Azithromycin group					
			Number of treatment response cases	Total number of cases	Dose	Frequency of administered doses	Route of administration	Duration of treatment
Wen-E Liu 2013 [10]	China	2013	13	90	1 g	qd	po	7d
Skerk 2000 [11]	Croatia	2000	28	30	1 g	Once	po	1d
Skerk 2000 [11]	Croatia	2000	17	30	0.5 g	qd	po	6d
Škerk 2000 [12]	Croatia	2000	44	50	0.5 g	qd	po	6d
Fatih Sendag 2000 [13]	Turkey	2000	15	21	1 g	once	po	1d
Steingrimssoir Olafnr 1990 [14]	USA	1990	11	14	1 g	once	po	1d
Steingrimssoir Olafnr 1990 [14]	USA	1990	5	5	500 mg d1, 250 mg d2–3	qd	po	3d

Study	Region	Year	Control group						
			Number of treatment response cases	Total number of cases	Drug	Dose	Frequency of administered doses	Route of administration	Duration of treatment
Wen-E Liu 2013 [10]	China	2013	20	98	Chinese Traditional Medicine	10 g	tid	po	14d
Skerk 2000 [11]	Croatia	2000	28	30	Doxycycline	100 mg	bid	po	7d
Skerk 2000 [11]	Croatia	2000	28	30	Doxycycline	100 mg	bid	po	14d
Škerk 2000 [12]	Croatia	2000	47	64	Doxycycline	100 mg	bid	po	7d
Fatih Sendag 2000 [13]	Turkey	2000	17	22	Doxycycline	100 mg	bid	po	7d
Steingrimssoir Olafnr 1990 [14]	USA	1990	9	12	Doxycycline		bid	po	1d
Steingrimssoir Olafnr 1990 [14]	USA	1990	12	16	Doxycycline		bid	po	7d

### Efficacy of azithromycin treatment

**Fig. 2:**
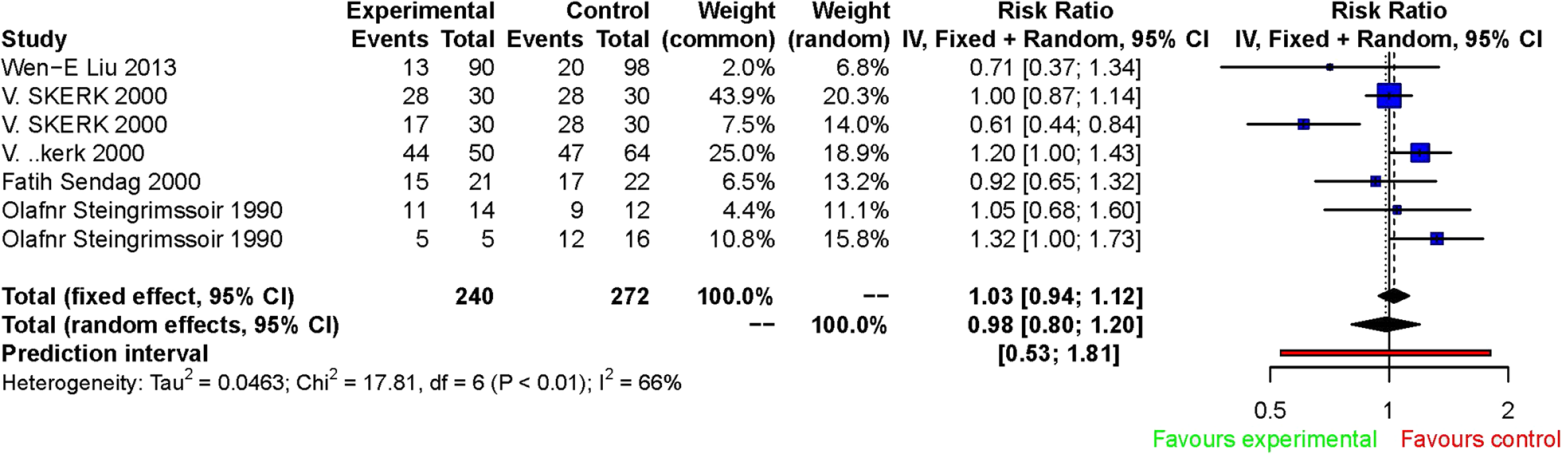
Forest plot of the efficiency of azithromycin treatment for *Ureaplasma urealyticum* infection

The primary outcome suggested no superiority in treating *Ureaplasma urealyticum* in the female reproductive tract with other antibiotics. Compared to other antibiotics, azithromycin has a comparable therapeutic effect on *Ureaplasma urealyticum* infection. The fixed effect of RR was 1.03 [0.94, 1.12], while the random effect of RR was 0.98 [0.80, 1.20] (Fig. 2).

### Subgroup analysis

**Fig. 3:**
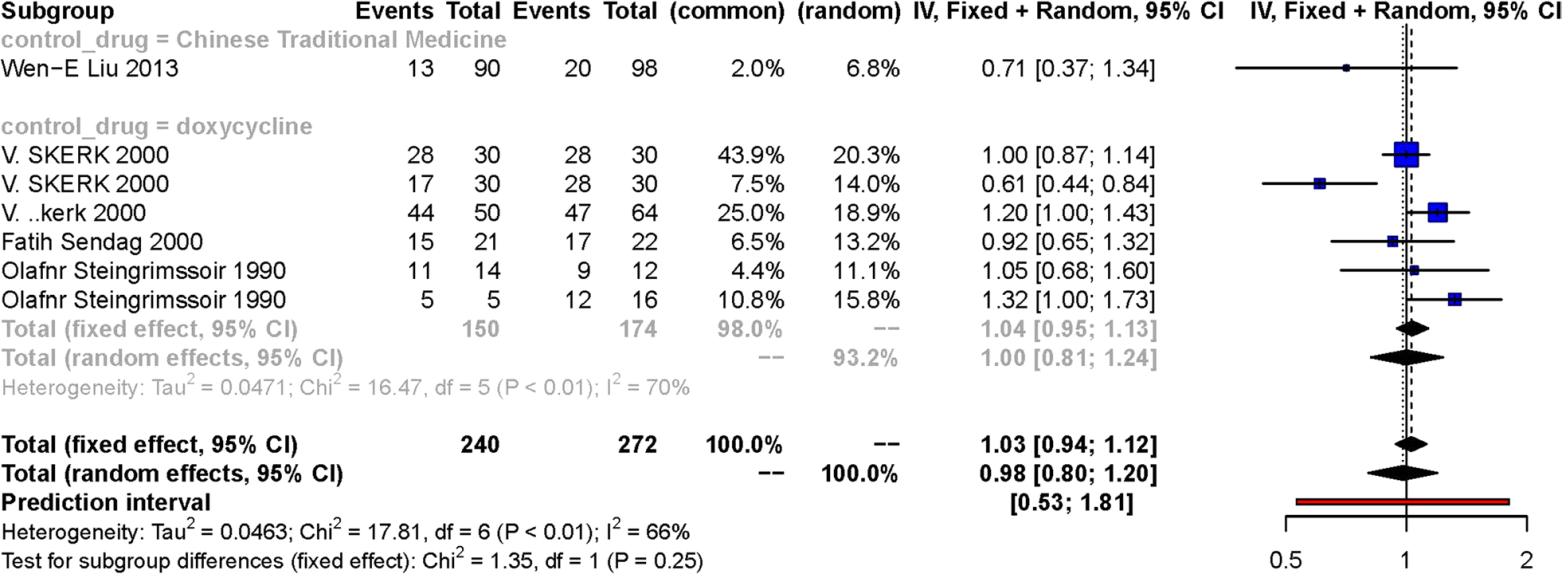
Forest plot of the efficiency of azithromycin treatment for *Ureaplasma urealyticum* infection versus Chinese traditional medicine arm and doxycycline arm

**Fig. 4:**
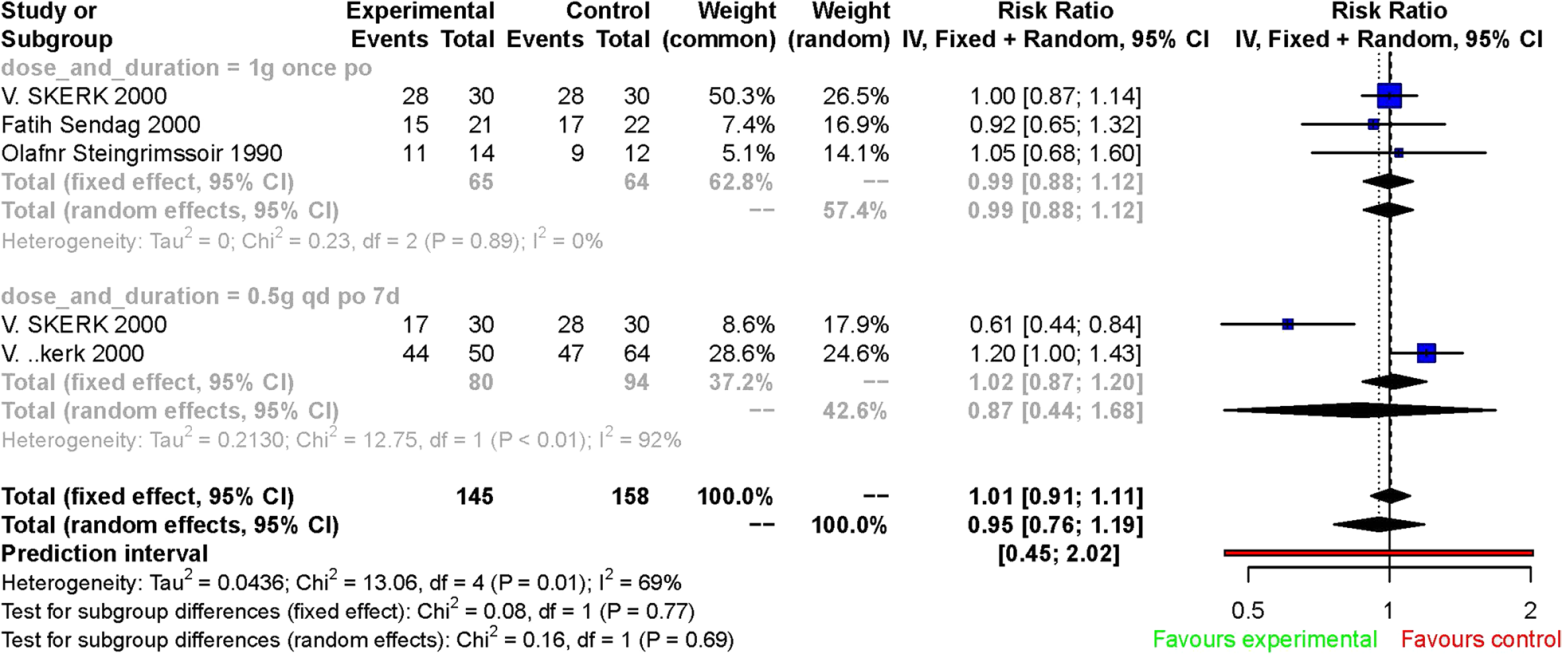
Forest plot of the efficiency of azithromycin treatment for *Ureaplasma urealyticum* infection in a single dose of 1 g arm and 0.5 g QD for 7 days arm

Subgroup analysis was performed according to the therapy drug in control group and dose and duration of azithromycin. When we divided the included RCTs into subgroups according to the drug prescribed in control group, we came to similar conclusions. Whether the control group was treated with doxycycline or traditional Chinese medicine, azithromycin had a similar efficacy. The fixed effect of RR was 1.04 [0.95, 1.13] (Fig. 3). We also considered the efficacy of azithromycin on *Ureaplasma urealyticum* at different doses and duration of administration, meta-analyses of the subgroup showed that they had a comparable therapeutic effect compared to controls, whether given as a single dose of 1 g or 0.5 g QD for 7 days. The fixed effect of RR in subgroup of a single dose of 1 g was 0.99 [0.88, 1.12] and in subgroup of 0.5 g QD for 7 days was 1.02 [0.87, 1.20] (Fig. 4).

### Quality assessment and risk of bias

**Fig. 5:**
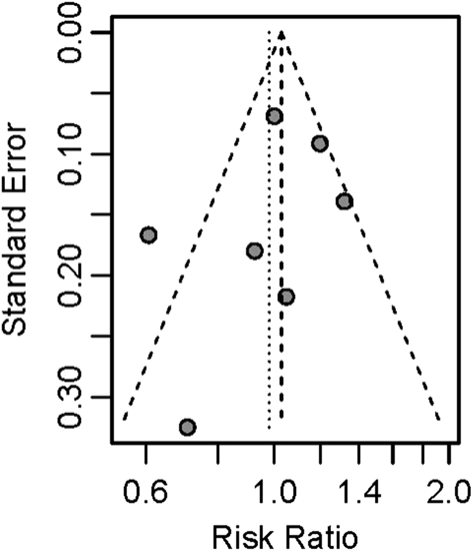
Funnel plot of the publication bias of azithromycin treatment efficiency

**Fig. 6:**
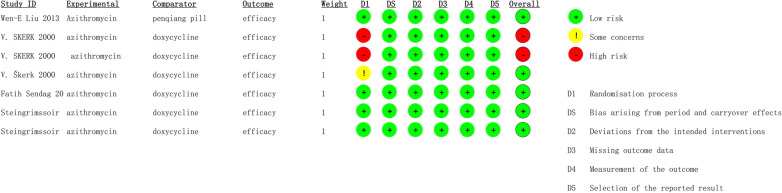
Results of the risk of bias assessment using RoB2

**Fig. 7:**
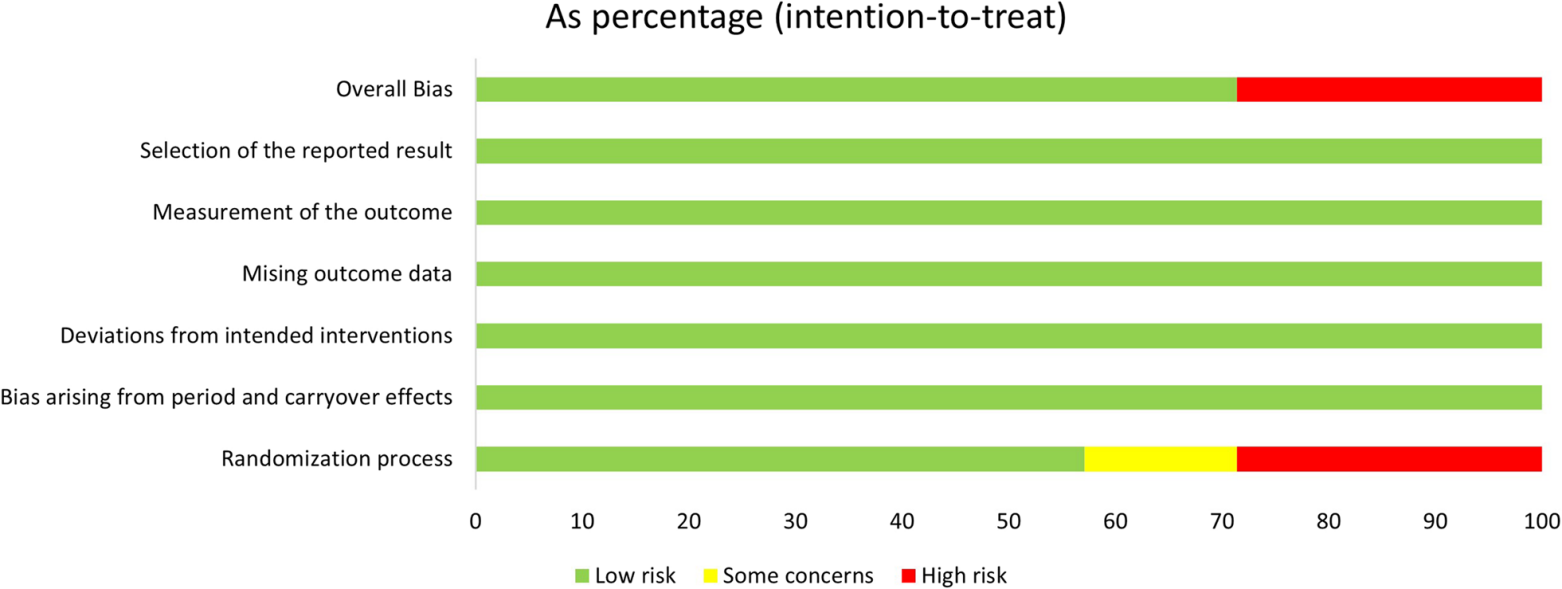
Bar chart overview and per-study risk of bias rating for RCT studies

Publication bias was tested for azithromycin treatment efficiency, as displayed in Fig. 5, and the funnel plot demonstrated good symmetry. Egger’s test showed no obvious publication bias in the rate of azithromycin treatment efficiency (p = 0.297). Risk of bias in randomized trials was examined with the Cochrane Risk of Bias 2 (RoB2) tool. According to RoB2, two studies were rated as having a high risk of bias. Other five studies were rated as having a low risk of bias. The detailed results of the risk of bias analyses are available in Figs. 6 and 7.

## Discussion

Recently, the prevalence of cervical mycoplasma infection has been increasing yearly [15, 16]. Mycoplasma cervicalis infection can cause a series of complications, including infertility, spontaneous abortion, pelvic inflammatory disease, and ectopic pregnancy, posing a great threat to women’s health during their reproductive years. In addition, habitual abortion, frequent sexual intercourse, and childbirth may also damage the cervix, causing pathogens to invade and form inflammation and, ultimately, induce cervicitis [6, 17]. Mycoplasma is the main pathogen that causes cervicitis and does not usually invade the bloodstream but rather often binds to the epithelial cells of the genitourinary tract or respiratory tract through adhesion and damages the cells via different mechanisms of action [18]. Some studies [16, 19] have reported that *Ureaplasma urealyticum* infection is the most prevalent in patients with cervicitis, followed by *Ureaplasma urealyticum* + mycoplasma hominis infection, whose infection rate is the lowest in mycoplasma hominis. The reason for this may be that *Ureaplasma urealyticum* is by far the simplest and smallest cell with the ability to reproduce itself, making it easier to invade a damaged cervix and cause infection compared to other pathogens [20]. Mycoplasma has no cell wall; thus, antibacterial drugs used for a cell wall are ineffective in its treatment.

At this stage, clinical treatment of mycoplasma is based on drugs such as quinolones, tetracyclines, macrolides, etc. The sensitivity of different mycoplasmas to different drugs is not exactly the same, and in vitro drug sensitivity tests also often have poor clinical application; therefore, the choice of antibiotics has not formed the first-line recommendation. In previous studies, it has been clearly stated that quinolones should not be used for empirical treatment of *Ureaplasma urealyticum* infection. Due to the high frequency of clinical use of ciprofloxacin and levofloxacin in recent years, *Ureaplasma urealyticum* is prone to developing resistance to them [21]. The resistance mechanism is mainly manifested by mutations in chromosomal DNA pro-rotase and topoisomerase IV, which are maintained in a highly resistant, stable state. Antibiotics containing β-lactamases are also ineffective against *Ureaplasma urealyticum* infection because they have no cell wall structure.

Azithromycin, a macrolide that interferes with cellular protein synthesis, is often used to treat patients with mycoplasma cervicitis. Azithromycin is well absorbed orally and has a wide tissue distribution. When azithromycin enters the body, the active drug is transported by macrophages to the site of infection and acts to inhibit anaerobic bacteria and mycoplasma for a long time. In addition, as far as drug safety is concerned, according to the classification of the drug look-up table for pregnancy risk class issued by the FDA, macrolides belong to class B. They provide better therapeutic safety for women of childbearing age and during pregnancy, as well as avoiding adverse pregnancy outcomes that should be caused by drug treatment [22].

This meta-analysis demonstrated that azithromycin has a positive therapeutic effect on *Ureaplasma urealyticum* infection and there is no correlation with the dose of azithromycin, whether it is a single dose of 1 g or 0.5 g QD for 6 days. Compared with the control group, two subgroups both showed comparable efficacy. However, a meta-analysis of the safety of azithromycin for *Ureaplasma* infection could not be conducted due to the limited amount of relevant literature as well as the lack of safety studies in some of the included studies. Previous research has generally indicated that azithromycin has a favorable safety profile in various treatment situations.

The present study followed PRISMA guidelines to ensure the quality of its methodology. The reliability of study results was also assessed using GRADE grading method. However, there are limitations to this study that should be identified. These include a small amount of included literature and insufficient sample size, the potential for language bias due to the inclusion of only English literature in the search strategy, and the need for larger, multi-regional, multi-center, randomized, double-blind clinical trials to validate the findings.

## Conclusion

Regarding the meta-analysis that we performed based on existing clinical studies, azithromycin has a comparable therapeutic effect on *Ureaplasma urealyticum* infection in the female reproductive tract compared to other antibiotics.

## Supplementary Information

**Additional file 1.**

## Data Availability

The datasets used and/or analysed during the current study are available from the corresponding author on reasonable request.

## Abbreviations

RCTs: Randomized controlled trials
INPLASY: International Platform of Registered Systematic Review and Meta-analysis Protocols
PRISMA: Preferred Reporting Items for Systematic Reviews and Meta-Analyses
MeSH: Medical Subject Headings
NOS: Newcastle–Ottawa Scale
OR: Odds ratio
CI: 95% Confidence interval
