# Supplementary material for: Efficacy of azithromycin in treating Ureaplasma urealyticum: a systematic review and meta-analysis
Source: BMC Infect Dis. 2023 Mar 16;23:163. doi: 10.1186/s12879-023-08102-5 (PMC10021952; doi:10.1186/s12879-023-08102-5)
Supplement: Supplementary file 1 — Additional file 1. [file 12879_2023_8102_MOESM1_ESM.docx]

Supplementary Material

Efficacy of azithromycin in treating ureaplasma urealyticum: a systematic review and meta-analysis

**Weibin Fan1,6 †, Qisheng Wang2†, Zuyu Liang3, Jinyu Wang4, Lin Zhang5***

*** Correspondence:** ZhangLin: zhanglinfudan@zju.edu.cn

**Retrieval search strategy:**

The PubMed search is as follows:

((((Ureaplasma urealyticum[MeSH Terms]) OR (Ureaplasma urealyticum biovar 2[Title/Abstract])) OR (Ureaplasma urealyticum[Title/Abstract]) AND (1865/1/1:2022/10/1[pdat])) AND ((azithromycin[MeSH Terms]) OR (azithromycin[Title/Abstract] OR Azythromycin[Title/Abstract] OR Sumamed[Title/Abstract] OR Toraseptol[Title/Abstract] OR Vinzam[Title/Abstract] OR CP-62993[Title/Abstract] OR CP 62993[Title/Abstract] OR CP62993[Title/Abstract] OR Zithromax[Title/Abstract] OR Azitrocin[Title/Abstract] OR Azadose[Title/Abstract] OR Ultreon[Title/Abstract] OR Zitromax[Title/Abstract] OR Azithromycin Dihydrate[Title/Abstract] OR Dihydrate, Azithromycin[Title/Abstract] OR Azithromycin Monohydrate[Title/Abstract] OR Monohydrate, Azithromycin[Title/Abstract] OR Goxal[Title/Abstract] OR Zentavion[Title/Abstract]) AND (1865/1/1:2022/10/1[pdat]))) AND (((randomized controlled trial[pt] OR controlled clinical trial[pt] OR randomized[tiab] OR randomised[tiab] OR placebo[tiab] OR drug therapy[sh] OR randomly[tiab] OR trial[tiab] OR groups[tiab]) NOT (animals[mh] NOT humans[mh]))

Embase Session Results

.......................................................

No. Query Results Results Date

#9. #7 AND 'human'/de AND 'randomized controlled 20 26 Jun 2022

trial'/de

#8. #7 AND 'human'/de 326 26 Jun 2022

#7. #1 AND #6 381 26 Jun 2022

#6. #2 OR #5 49,336 26 Jun 2022

#5. #3 AND #4 888 26 Jun 2022

#4. 'azithromycin':ab,ti OR 'azithromycin 25,165 26 Jun 2022

monohydrate':ab,ti OR 'monohydrate' OR

'monohydrated' OR 'monohydrates' OR

'monohydration'

#3. 'dihydrate' OR 'dihydrated' OR 'dihydrates' 5,240 26 Jun 2022

#2. 'azithromycin'/exp OR 'azithromycin':ab,ti OR 48,499 26 Jun 2022

'sumamed':ab,ti OR 'cp-62993':ab,ti OR

'zithromax':ab,ti OR 'azitrocin':ab,ti OR

'azadose':ab,ti OR 'zitromax':ab,ti OR

'azithromycin dihydrate':ab,ti

#1. 'ureaplasma urealyticum'/exp OR 'ureaplasma 3,660 26 Jun 2022

urealyticum':ab,ti OR 'ureaplasma urealyticum

biovar 2':ab,ti

Cochrane Library

P

Ureaplasma urealyticum OR Ureaplasma urealyticum biovar 2

I

azithromycin or Azythromycin or Sumamed or Toraseptol or Vinzam or CP-62993 or CP 62993 or CP62993 or Zithromax or Azitrocin or Azadose or Ultreon or Zitromax or Azithromycin Dihydrate or Dihydrate, Azithromycin or Azithromycin Monohydrate or Monohydrate, Azithromycin or Goxal or Zentavion

S

randomized controlled trial OR controlled clinical trial OR random allocation OR double-blind OR single-blind OR placebo OR Randomly OR randomized OR clnical trial* OR trial* OR RCT OR Random*

Web of Science

((TS=(Ureaplasma urealyticum or Ureaplasma urealyticum biovar 2)) AND TS=(Azithromycin or Azythromycin or Sumamed or Toraseptol or Vinzam or CP-62993 or CP 62993 or CP62993 or Zithromax or Azitrocin or Azadose or Ultreon or Zitromax or Azithromycin Dihydrate or Dihydrate, Azithromycin or Azithromycin Monohydrate or Monohydrate, Azithromycin or Goxal or Zentavion)) AND TS=(randomized controlled trial OR controlled clinical trial OR random allocation OR double-blind OR single-blind OR placebo OR Randomly OR randomized OR clnical trial* OR trial* OR RCT OR Random*)
